# A Linear Regression Predictor for Identifying N^6^-Methyladenosine Sites Using Frequent Gapped K-mer Pattern

**DOI:** 10.1016/j.omtn.2019.10.001

**Published:** 2019-10-10

**Authors:** Y.Y. Zhuang, H.J. Liu, X. Song, Y. Ju, H. Peng

**Affiliations:** 1 School of Informatics, Xiamen University, Xiamen 361005, China; 2 College of Information Technology and Computer Science, University of the Cordilleras, Baguio 2600, Philippines; 3 School of Computer and Information Technology, Nanyang Normal University, Nanyang 473000, China

**Keywords:** N6-methyladenosine, RNA modifications, novel feature extraction algorithm, frequent gapped k-mer pattern, linear regression, Saccharomyces cerevisiae database, 10-fold cross-validation, genome analysis

## Abstract

N6-methyladenosine (m^6^A) is one of the most common and abundant modifications in RNA, which is related to many biological processes in humans. Abnormal RNA modifications are often associated with a series of diseases, including tumors, neurogenic diseases, and embryonic retardation. Therefore, identifying m^6^A sites is of paramount importance in the post-genomic age. Although many lab-based methods have been proposed to annotate m^6^A sites, they are time consuming and cost ineffective. In view of the drawbacks of the intrinsic methods in RNA sequence recognition, computational methods are suggested as a supplement to identify m^6^A sites. In this study, we develop a novel feature extraction algorithm based on the frequent gapped k-mer pattern (FGKP) and apply the linear regression to construct the prediction model. The new predictor is used to identify m^6^A sites in the *Saccharomyces cerevisiae* database. It has been shown by the 10-fold cross-validation that the performance is better than that of recent methods. Comparative results indicate that our model has great potential to become a useful and effective tool for genome analysis and gain more insights for locating m^6^A sites.

## Introduction

Over 100 modifications occur in RNA.^1^ The functions of internal modifications of mRNA are used to keep the stability of mRNA, and the most common internal modifications of mRNA include N^6^-methyladenosine (m^6^A), N^1^-methyladenosine (m^1^A), 5-methylcytosine (m^5^C). Among them, global scientists have verified many enzymes that m^6^A engages, such as histone demethylases, methylase, and methylation recognition enzyme.^2^ Abnormal m^6^A modifications are often related to a series of diseases, including tumors, neurogenic diseases, and embryonic retardation.^3^ RNA m^6^A was first observed in 1970s.^4^ Since then, m^6^A is found in a wide spectrum of all living organisms and linked to many important roles of biological activities, including mRNA splicing, stability, nuclear processing, and immune response.^5^, ^6^, ^7^, ^8^ Therefore transcriptome-wide annotation of m^6^A sites will be helpful to understand its biological functions.

In the past few years, high-throughput sequencing techniques such as MeRIPSeq^9^ and m^6^A-seq^10^ have identified m^6^A peaks in *Saccharomyces cerevisiae*, *Mus musculus*, and *Homo sapiens*. At the same time, the miCLIP technique^11^ was proposed to provide the recognition method of m^6^A sites in the human transcriptome. However, in consideration of the biological inherent reliance of the techniques,^12^ they are still neither budget nor time efficient in performing transcriptome-wide analysis.

Although lab-based technologies have been widely applied to identify m^6^A, some cost-effective computational methods are developed in assisting the process as well. To identify methylated m^6^A sites, building a high-resolution database is of paramount importance in predicting m^6^A sites. Using the high-resolution database of *Saccharomyces cerevisiae* constructed by Schwartz et al.,^13^ Chen et al.^14^, ^15^, ^16^, ^17^, ^18^ proposed a series of predictors such as “iRNA-Methyl,” “M6ATH,” “MethyRNA,” “iRNA-3typeA” and “iRNA(m6A)-PseDNC,” which formulated RNA sequences by using different combinations of feature extractions and classifiers to make predictions. Feng et al.^19^ used a method called “iRNA-PseColl,” which incorporated collective features of the RNA sequence elements into PseKNC to make predictions. Jaffrey et al.^11^ built a single-nucleotide resolution map of m^6^A sites across *Homo sapiens*. More recently, Chen et al.^20^ proposed a support-vector-machine-based method to predict m^6^A sites in *Arabidopsis thaliana*. As mentioned in some references, well-established ensemble classifiers have been proven to outperform single classifiers.^21^, ^22^, ^23^ Based on this, Wei et al.^24^ thus proposed an m^6^A predictor by constructing an ensemble classifier based on the support vector machine (SVM) to successfully improve the predictive performance. Wei et al.^25^^,^^26^ have also done a lot of research with the ensemble classifier, which has great significance for reference in our study.

In this article, we propose a novel method for the identification of m^6^A sites within RNA sequences. As for feature representation, we use the frequent gapped k-mer pattern (FGKP) discovery algorithm to mainly capture the properties in RNA sequences. In the predictive model, we use the linear regression to discriminate the positive and negative samples. Experimental results show that our model outperformed other existing methods in the literature under the 10-fold cross-validation test.

## Results

Several diseases have their underlying causes in RNA,^27^^,^^28^ including cancers.^29^, ^30^, ^31^ In our study, we combined the advantage of effective extraction of frequent gapped k-mer (FGK) and the strong ability of classification of the linear predictive model to create a powerful predictive tool in order to discriminate the positive and negative samples of m^6^A. The learning machine that we used was logistic regression (LR). We have experimented with our predictor in the *Saccharomyces cerevisiae* genome using 10-fold cross-validation. It turns out that our model is superior to M6A-HPCS, the recent classifier in this area, and also has a better performance than other feature extractions and different parameters within our model. We anticipate that it will shed some light on genome analysis in future practice.

### Four Evaluation Metrics

In general, the following four metrics are used to measure the quality of a predictor:^32^ sensitivity (*SN*), specificity (*SP*), accuracy (*ACC*), and Matthews correlation coefficient (*MCC*). These metrics were first introduced by Chou^33^ and then they were widely applied to a wide range of biological areas (see Liu et al.,^34^, ^35^, ^36^, ^37^ Ehsan et al.,^38^ Feng et al.,^19^ Song et al.,^39^ Lin et al.,^40^ and Xu et al.^40^^,^^41^). Their definitions are as follows:

(Equation 1) $$\mathrm{SP}=\frac{\mathrm{TN}}{\mathrm{TN}+\mathrm{FP}}\times 100\%$$

(Equation 2) $$\mathrm{SN}=\frac{\mathrm{TP}}{\mathrm{TP}+\mathrm{FN}}\times 100\%$$

(Equation 3) $$\mathrm{ACC}=\frac{\mathrm{TP}+\mathrm{TN}}{\mathrm{TP}+\mathrm{FN}+\mathrm{TN}+\mathrm{FP}}\times 100\%$$

and

(Equation 4) $$\mathrm{MCC}=\frac{\mathrm{TP}\times \mathrm{TN}-\mathrm{FP}\times \mathrm{FN}}{\sqrt{\left(\mathrm{TP}+\mathrm{FN}\right)\left(\mathrm{TN}+\mathrm{FP}\right)\left(\mathrm{TP}+\mathrm{FP}\right)\left(\mathrm{TN}+\mathrm{FN}\right)}}$$

where *TP*, *TN*, *FP*, and *FN* are true positive, true negative, false positive, and false negative, respectively. In this research, *TP* represents the true m^6^A site predicted correctly, *TN* represents the non-m^6^A site predicted incorrectly, *FP* represents the non-m^6^A site predicted incorrectly as the true m^6^A site, and *FN* represents the non-m^6^A site predicted correctly as the non-m^6^A site. The values of *SN*, *SP*, *ACC*, are between 0 and 1. The closer to 1 they get, the more accuracy our model achieves; the value of *MCC* is between −1 and 1. The larger the value that *MCC* gets, the better performance our prediction model obtains.

### Cross-Validation

Normally, three types of validation are used to derive the metric values: independent test sets, subsampling (or K-fold cross-validation), and the jackknife test (or LOOCV). Although the jackknife test can fully train the data we already have to acquire a more accurate classifier, and it has definite sampling and error estimation based on the specific dataset, the jackknife test is not a time-efficient method compared with the other two types of validation. In this article, we adopted the 10-fold cross-validation method used by many researchers^42^, ^43^, ^44^ in this area.

### ROC Curve

ROC curve (also called the sensitivity curve) is the abbreviation for receiver operating characteristic curve. Every point on the curve reflects the same sensitivity. They react to the same signal simulation in the different judgment standards. Therefore, the ROC curve can be generally treated as the overall performance in the binary classification problems. The ROC curve is normally plotted with the x-axis true-positive rate (TPR) and the y-axis false-positive rate (FPR) in the different thresholds of the classification. We can understand the TPR as the sensitivity as described earlier, and the FPR can be computed as 1 − specificity. The area under the ROC curve (AUROC) can also be calculated. The AUROC is the indicator of the performance of a predictor. The AUROC ranges from 0.5 to 1. The closer the AUROC score of a predictor to 1, the better and more robust the predictor we can reckon, and we can deem the AUROC score of 0.5 of a predictor as a random predictor.

## Discussion

### Comparison among Different Feature Extractions

**Figure 1:**
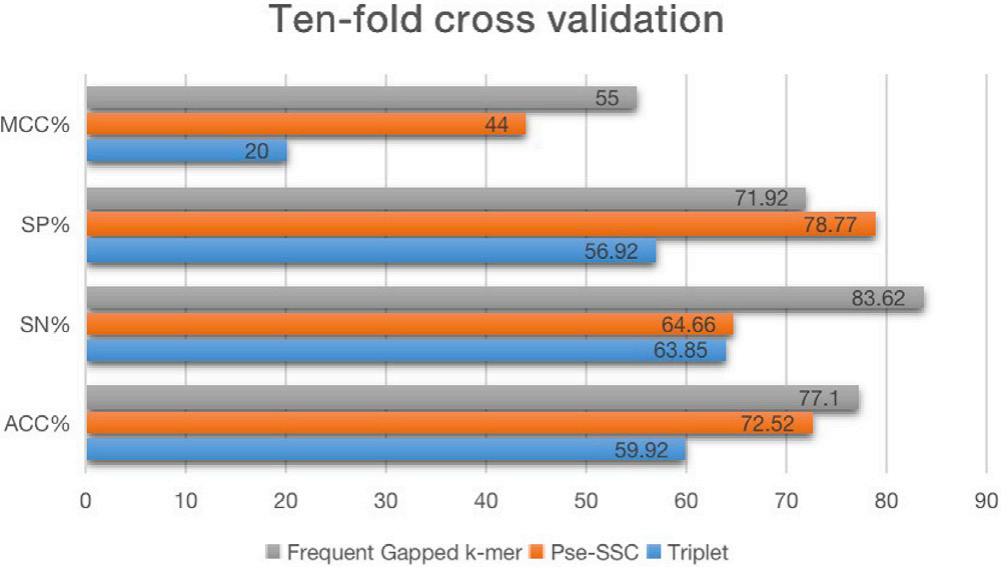
Performance of Different Feature Extractions Using 10-Fold Cross-validation Here, we compare the effect of our feature extraction (FGK) with Pse-SSC and Triplet methods.

**Figure 2:**
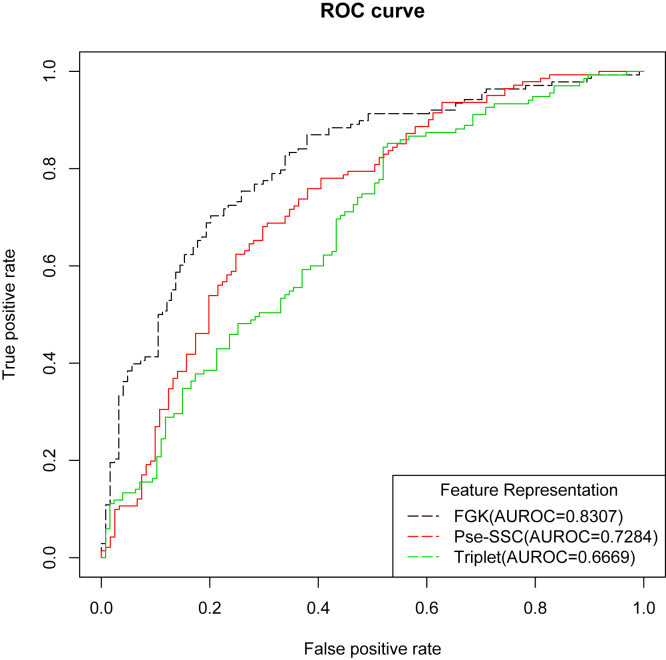
ROC Curves of Frequent Gapped K-mer, Pse-SSC, and Triplet and Their AUROC Values

To justify our feature extraction technique, we make comparisons with two of the most commonly used feature representation techniques, Triplet and Pse-SSC, and this shows that the FGK method gets the much better performance than the other two feature representations. We show the result in Table 1, and from Figure 1, we can see the graphical comparisons from four different evaluation metrics. The FGK leads Pse-SSC by 4% and Triplet by 17% for the *ACC*, and for the *MCC* metric, FGK outnumbers its counterparts by over 10%. From Figure 2, we can see the effects of three different feature extractions from their ROC curves. The larger areas under the curve we get, the better performance the method achieves. Also, we can also see from Table 1 that our feature representation is 63.2% and 16.4% higher than features Pse-SSC and Triplet, respectively.

**Table 1:** Comparison of Different Feature Extractions

Feature	SP (%)	SN (%)	ACC (%)	MCC	AUROC
Triplet	56.92	63.85	59.92	0.20	0.6669
Pse-SSC	78.77	64.66	72.52	0.44	0.7284
Frequent gapped k-mer	71.92	83.62	77.10	0.55	0.8307

### Comparison with Other Classifiers

**Figure 3:**
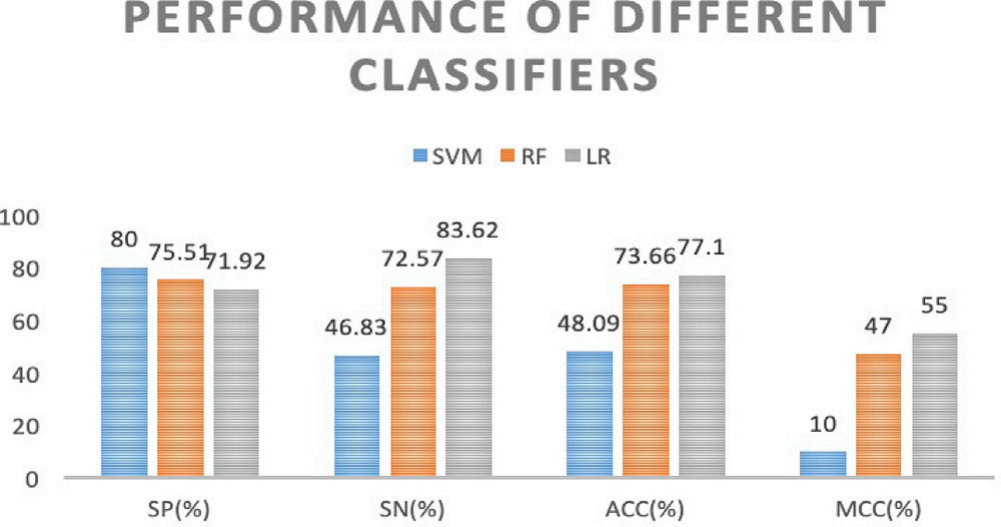
Comparison of Performances among the LR Classifier and Other Popular Classifiers (SVM and RF) with the Same Learning Feature Representations on the *S. cerevisiae* Dataset

In Table 2, we compare LR with SVM and random forest (RF). The reason for choosing SVM and RF for comparison is because SVM^20^^,^^21^^,^^45^^,^^46^ and RF^5^^,^^47^, ^48^, ^49^, ^50^ are two of the most widely used classifiers in bioinformatics. Although the *SP* of the proposed method is lower than those of SVM and RF, its *SN*, *ACC*, and *MCC* are higher than those of SVM and RF, indicating that the performance of the LR-based model can effectively discriminate the m^6^A sites in *Saccharomyces cerevisiae*. We can see the overall performance of three classifiers in Figure 3. In this figure, we can see that, although the *SP* of LR performs poorly compared to that of the other two classifiers, the other three metrics are much better than the rest for the two predictors. The *ACC* of LR is far better than that of SVM, topping by almost 30% and slightly exceeding by 3.5% the *ACC* of RF.

**Table 2:** Performance Comparison of Different Classifiers

Classifier	SP (%)	SN (%)	ACC (%)	MCC
SVM	80	46.83	48.09	0.10
RF	75.51	72.56	73.66	0.47
LR	71.92	83.62	77.10	0.55

### Comparison with Different Parameters

In Table 3, we compared the model prediction performance of linear regression using different parameters and found that, with parameters *k* = 4 and γ = 0.025, we get the most desirable result. The classifier with parameters *k* = 5 and γ = 0.05 is almost 25% higher than its counterpart in *ACC*.

**Table 3:** Performance Comparison of Different Parameters in Our Model

Classifier	SP (%)	SN (%)	ACC (%)	MCC
LR (k = 5, γ = 0.05)	73.53	73.81	73.66	0.47
LR( k = 4, γ = 0.025)	71.92	83.62	77.10	0.55

### Comparison with Existing Predictors

To evaluate the performance of our proposed predictor, we compared our predictor with two existing predictors, iRNA-Methyl^14^ and M6A-HPCS.^51^ The reason to choose these two predictors for comparison is that they have been reported to achieve outstanding performance in m^6^A site identification. For fairness of comparison, all compared predictors are trained and validated on the same benchmark dataset. The results are summarized in Table 4. It can be observed that, among the compared predictors, the proposed model obtains the best performance in terms of *ACC* and *MCC*, with 77.10% and 55%, respectively. Compared with the best of the existing predictors, M6A-HPCS, our classifier performance is about 10% higher for *ACC* and 20% higher for *MCC*.

**Table 4:** Comparison of M6APred-FG with Other Well-Known Classifiers

Prediction Method	SP (%)	SN (%)	ACC (%)	MCC
iRNA-Methyl	60.63	70.59	65.59	0.29
M6A-HPCS	62.89	71.77	67.33	0.35
iRNA-Freq	71.92	83.62	77.10	0.55

## Materials and Methods

### Framework of the Proposed Predictor

**Figure 4:**
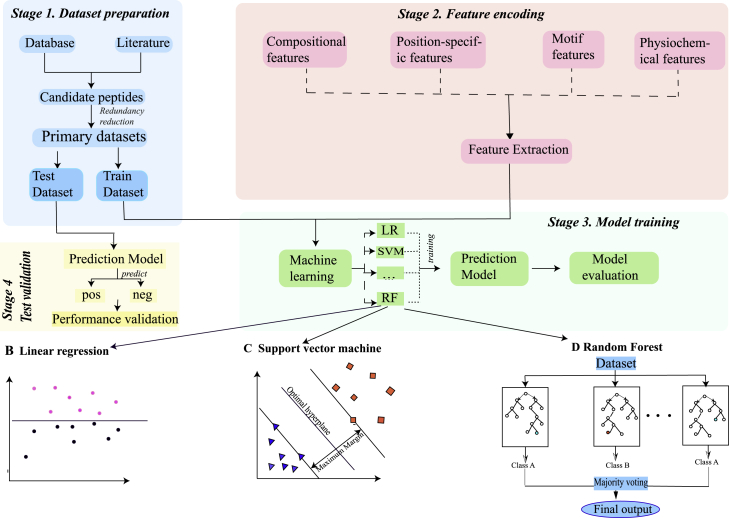
Flowchart of the Proposed Predictor Stage 1 shows the procedure of dataset preparation. We chose a benchmark database and used updated literature to obtain candidate peptides. Since the candidate peptides have imbalanced positive and negative samples, we needed to balance the samples (or reduce redundancy) to get the primary dataset. Then, we divided the dataset into the test dataset and the train dataset. Stage 2 shows the feature encoding or feature extraction. In our sample sequences, there is information hidden. We needed to find a way to extract their features to best represent the original samples and digitalize them. Stage 3 shows how we used the train dataset and chose the appropriate model to gain a prediction model and evaluate it. Stage 4 shows how we tested and validated our predictive model. In our article, we combined stages 3 and 4 together using 10-fold cross-validation to evaluate our model.

Figure 4 shows the flowchart of the proposed predictor. The first stage is to collect data from verified databases and relevant literature.^14^^,^^15^^,^^52^ In this research, we use the organized dataset from Chen et al.’s^14^ work. The second stage is feature encoding. This stage includes feature representation and feature optimization. Feature representation means extracting characteristics of RNA sequences using various feature descriptors, including composition features like Dinucleotide-based auto covariance (DAC), physicochemical features like PC-PseDNC-General, and our newly found FGKP. The final stage is to train the machine learning model (i.e., SVM, RF, and linear regression) using the feature extraction from the last stage. The predictive model constructed is based on the feature extraction mentioned earlier and validated through validation methods. In this study, we used the 10-fold cross-validation test.

### Datasets

m^6^A sites have been widely identified in *Saccharomyces cerevisiae*,^13^ *Homo sapiens*,^10^^,^^11^ *Mus musculus*,^10^ and *Arabidopsis thaliana*.^53^ In this work, we used the dataset from *Saccharomyces cerevisiae*. In the *Saccharomyces cerevisiae* genome, m^6^A sites have the same motif, GAC, and they are more easily methylated.^13^ Since RNA sequences in *Saccharomyces cerevisiae* have different lengths, we used the organized dataset from Chen et al.’s^14^ work. There are 1,307 positive samples and 1,307 negative samples, where the negative samples were randomly collected from 33,280 sequences with non-m^6^A sites. All sequences in the dataset are 51 nt long (25 nt on each side of the m^6^A/non-m^6^A sites), with the sequence similarity less than 85%.

### Representation of RNA Sample

The RNA samples in our dataset can be generally expressed as the following pattern:

(Equation 5) $$R=M_{1}M_{2}M_{3}\cdots M_{51}$$

where

$M_{i}\in \left\{A\left(adenine\right),C\left(cyto\;\sin\;e\right),G\left(guanine\right),U\left(uracil\right)\right\}i=1,2,3,\cdots ,51.$

The first thing we would need to do is to transform the RNA sequence in Equation 5 to a vector. However, a vector might lose its sequential information and pattern. In order to solve the problem, we introduce the FGKP discovery algorithm that we recently found. In this method, we can separate our algorithm into four steps and elaborate each step accordingly:

(1) Search all the FGK sub-sequences from each sequence in the dataset.

We find all FGK sub-sequences from each sequence in the dataset and calculate the frequency of gapped k-mer sub-sequences, and we can set the frequency threshold here. Here, the parameter *k* means the matching length of the sub-sequences, and we denote the frequency threshold as γ.

(2) Build a set for the frequent sub-sequences.

**Figure 5:**
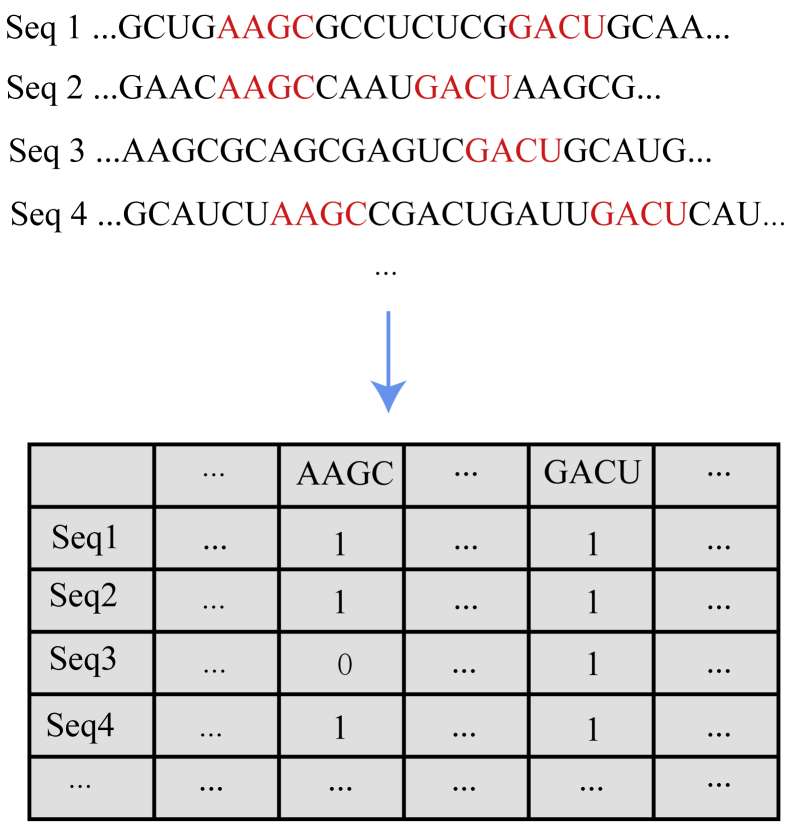
The Transformation from the Original Samples to 0–1 Sequences

FGK are subjects and whose lengths over a threshold is an attribute clause which modifies the subjects. We can map each FGK sub-sequence into a column of the table as shown in Figure 5.

(3) Utilize the frequent k-mer sub-sequence set as features to generate vectors.

First of all, we define the following functions:

(Equation 6) $$c\left(S_{i},FkM_{j}\right)=\{\begin{array}{cc} 1, & if\;FkM_{j}\;exactly\;matches\;S_{i}\\ 0, & Otherwise \end{array}$$

(Equation 7) $$\phi \left(S_{i}\right)=\left(\begin{array}{c} c\left(S_{i},FkM_{1}\right)\\ c\left(S_{i},FkM_{2}\right)\\ \begin{array}{c} \ldots \\ c\left(S_{i},FkM_{n}\right) \end{array} \end{array}\right).$$

Here, ${\text{S}}_{\text{i}}$ denotes the sequence that is predicted, and ${\text{FkM}}_{\text{j}}$ denotes the j-element of the frequent k-mer sub-sequence set. As you can see from the function in Equation 6, we define a function c, which compares the predicted sequence ${\text{S}}_{\text{i}}$ and the j-element of the frequent k-mer sequence set, and we discriminate the perfect matching between ${\text{S}}_{\text{i}}$ and ${\text{FkM}}_{\text{j}}$ using 1 and 0 otherwise. After this procedure, we map the sequence ${\text{S}}_{\text{i}}$ using the function ϕ to a 0–1 vector as shown in the function in Equation 7.

### Linear Predictive Model

Although a huge amount of literature is related to classification methods such as SVM^21^^,^^52^^,^^54^, ^55^, ^56^, ^57^, ^58^, ^59^, ^60^, ^61^, ^62^ and RF,^5^^,^^47^, ^48^, ^49^, ^50^ as we can see from the feature representation algorithm of RNA sample, a series of sparse data is produced. Therefore, the need to deal with a large amount of sparse data is imperative. The linear predictive model is a linear classifier for processing a large amount of sparse data with a large number of examples and features. It is a general term for supervised models, including LR, SVM, and support vector regression (SVR). In this study, we used the packages LIBSVM^63^ and LIBLINEAR.^64^ They support the multiple types of linear classifiers that we mentioned earlier. In this study, we used LR and achieved a good result. LR uses the optimal decision boundary to construct regression formula and fitted parameter sets. The main idea is as follows:

1. Construct the prediction function ${\text{h}}_{\text{θ}}$, where θ represents the parameter sets of eigenvalue X.

**Figure 6:**
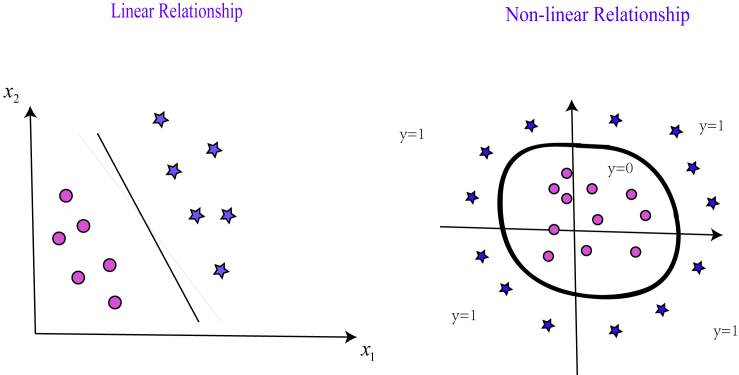
The Linear and Non-linear Relationships between ${\text{h}}_{\text{θ}}$and X For details, see the Linear Predictive Model section in Materials and Methods.

As far as we know, ${\text{h}}_{\text{θ}}$could have a linear relationship or non-linear relationship with X, as we can see from Figure 6. Normally, we can represent the linear relationship between ${\text{h}}_{\text{θ}}$and X using the formula

(Equation 8) $$h_{\theta }\left(x\right)=g\left(\theta_{0}+\theta_{1}x_{1}+\theta_{2}x_{2}\right)$$

and the non-linear relationship using the formula

(Equation 9) $$h_{\theta }\left(x\right)=g\left(\theta_{0}+\theta_{1}x_{1}+\theta_{2}x_{2}+\theta_{3}x_{1}^{2}+\theta_{4}x_{2}^{2}\right).$$

In linear programming, the idea of cost function is to minimize the difference of predictive result ${\text{h}}_{\text{θ}}$and actual y; i.e.,

(Equation 10) $$J\left(\theta \right)=\frac{1}{m}\sum_{i=1}^{m}\frac{1}{2}{\left(h_{\theta }\left(x^{\left(i\right)}\right)-y^{\left(i\right)}\right)}^{2}.$$

Then in LR, we can represent J(θ) as:

(Equation 11) $$\;J\left(\theta \right)=\frac{1}{m}\sum_{i=1}^{m}Cost\left(h_{\theta }\left(x^{\left(i\right)}\right),y^{\left(i\right)}\right).$$

2. Use gradient descent to calculate the maximum of J(θ).

We can achieve the maximum of J(θ) through fitting parameters using the gradient of the function. For simplicity, we can consider the following cost function

(Equation 12) $$J\left(\theta \right)=\frac{1}{m}\sum_{i=1}^{m}Cost\left(h_{\theta }\left(x^{\left(i\right)}\right),y^{\left(i\right)}\right)$$

(Equation 13) $$Cost\left(h_{\theta }\left(x\right),y\right)=\{\begin{array}{c} -\log\left(h_{\theta }\left(x\right)\right)\;\;\;if\;y=1\\ -\log\left(1-h_{\theta }\left(x\right)\right)\;\;if\;y=0 \end{array}$$

and we can renew the parameter ${\text{θ}}_{\text{j}}\text{:}={\text{θ}}_{\text{j}}+\text{α}\left(\partial \text{J}\left(\text{θ}\right)/\partial {\text{θ}}_{\text{j}}\right)$; that is,

(Equation 14) $$\theta_{j}:=\theta_{j}-\alpha \sum_{i=1}^{m}\left(h_{\theta }\left(x^{\left(i\right)}\right)-y^{\left(i\right)}\right)x_{j}^{\left(i\right)}.$$

## Author Contributions

Y.Z. conceived the project, designed the experiments, and edited the final version of the paper. H.L. performed the experiment. X.S. wrote the paper and drafted the figures. H.P. contributed to materials and data analysis.

## Conflicts of Interest

The authors declare no competing interests.
